# Intolerance of Uncertainty and Emotion Dysregulation as Predictors of Generalized Anxiety Disorder Severity in a Clinical Population

**DOI:** 10.3390/jcm14051502

**Published:** 2025-02-24

**Authors:** Sébastien Larochelle, Michel J. Dugas, Frédéric Langlois, Patrick Gosselin, Geneviève Belleville, Stéphane Bouchard

**Affiliations:** 1 Département de Psychologie et de Psychoéducation, Université du Québec en Outaouais, Gatineau, QC J8X 3X7, Canada; sebastien.larochelle@uqo.ca (S.L.); michel.dugas@uqo.ca (M.J.D.); 2 Département de Psychologie, Université du Québec à Trois-Rivières, Trois-Rivières, QC G8Z 4M3, Canada; frederic.langlois@uqtr.ca; 3 Département de Psychologie, Université de Sherbrooke, Sherbrooke, QC J1K 2R1, Canada; patrick.gosselin@usherbrooke.ca; 4 École de Psychologie, Université Laval, Québec, QC G1V 0A6, Canada; genevieve.belleville@psy.ulaval.ca

**Keywords:** generalized anxiety disorder, intolerance of uncertainty, emotion dysregulation, clinical population

## Abstract

**Background/objectives:** Several factors have been shown to play a role in the development and maintenance of generalized anxiety disorder (GAD), including intolerance of uncertainty and emotion dysregulation. Although the individual contribution of both of these factors is well documented, their combined effect has yet to be studied in a clinical population with GAD. The aim of the present study was to examine the relative contribution of intolerance of uncertainty and emotion dysregulation to the prediction of worry and GAD severity in adults with GAD. **Methods:** The sample consisted of 108 participants diagnosed with GAD. The participants completed measures of worry, GAD severity, depressive symptoms, intolerance of uncertainty, and emotion dysregulation. **Results:** Multiple regression indicated that both intolerance of uncertainty and emotion dysregulation significantly contributed to both worry and GAD severity, over and above the contribution of depressive symptoms. Of note, the model explained 36% of the variance in GAD severity scores. **Conclusions:** The present results provide preliminary evidence of complementarity among dominant models of GAD, and point to the potential role of integrative conceptualizations and treatment strategies for GAD.

## 1. Introduction

Generalized anxiety disorder (GAD) is a relatively common problem. Up to 9% of adults in the general population will suffer from it at some point in their lives [1,2,3,4,5,6,7]. Once present, GAD symptoms can last up to 20 years [8,9,10,11] and may persist even after psychotherapy [12,13,14]. Considering that residual symptoms may lead to significant consequences such as disturbances in functioning and reduced quality of life [15,16,17,18,19], there is a need to develop psychotherapies that are more effective for GAD. By furthering our understanding of the combined effects of factors contributing to the development and maintenance of GAD, we should be in a better position to develop more effective and comprehensive psychotherapeutic interventions.

Numerous models have been developed to clarify the etiology of GAD [20]. Of these, the Intolerance of Uncertainty Model (IUM) [21] has received particularly strong empirical support. According to the IUM, the core dysfunctional factor in GAD is intolerance of uncertainty, which was originally defined as “a relatively broad construct representing cognitive, emotional, and behavioral reactions to uncertainty in everyday life situations” [22] (p. 792). The role of intolerance of uncertainty in the development and maintenance of chronic anxiety and GAD has been repeatedly supported by empirical research, including the finding that intolerance of uncertainty is the component of the IUM that best predicts GAD severity. The IUM has been empirically supported in clinical populations. Research indicates that patients with GAD are more intolerant of uncertainty than are adults without GAD [21,23,24,25] or those who have various other anxiety disorders [25]. In addition, data indicate that the severity of intolerance of uncertainty, as measured by the Intolerance of Uncertainty Scale [22], is predictive of worry intensity [25] and severity of GAD [26]. Finally, treatment research shows that a reduction in the level of intolerance of uncertainty predicts a reduction in the severity of worry in patients receiving cognitive behavioral therapy for GAD [27,28]. In short, the current state of research supports the IUM and indicates that intolerance of uncertainty is associated with worry and GAD severity.

A second model of GAD that has received considerable attention is the Emotion Dysregulation Model (EDM) [29,30,31]. According to the EDM, GAD is associated with various difficulties regulating emotions. For example, the EDM suggests that chronic worry can be understood as a strategy to manage emotions that are experienced as intense, hard to understand, and difficult to control or suppress. Rather than paying attention to, understanding, and experiencing emotions, people with GAD use worry to avoid the distress associated with emotions. A number of studies, most often using the Difficulties in Emotion Regulation Scale (DERS) [32], have provided support for the EDM. These studies show that individuals with GAD report greater difficulty regulating their emotions than do non-anxious individuals [31,33,34,35] and people with other mental health disorders [36,37,38,39,40]. Several studies also show that deficits in emotion dysregulation predict the level of anxiety and other symptoms in non-clinical samples [31,36,37,39,41,42,43,44,45,46,47] and clinical samples [31,36]. Finally, it has been reported that an improvement in emotion dysregulation skills predicts a decrease in the severity of worry in patients receiving cognitive behavioral therapy for GAD [48,49]. Thus, the EMD has received significant empirical support as an etiological model of chronic worry and GAD.

Intolerance of uncertainty has been conceptualized in many different ways since the construct was first defined by Freeston et al. in 1994 [22]. Generally speaking, recent definitions have emphasized the cognitive, behavioral, and emotional components of intolerance of uncertainty (see [50]), with the emotional component referring to a difficulty enduring the aversive emotional response elicited by the unknown elements of a given situation. It may be that emotion dysregulation interacts with intolerance of uncertainty to heighten the aversive quality of the emotional response to uncertainty. Although intolerance of uncertainty and emotion dysregulation have each received considerable support as vulnerability factors for worry and GAD, their respective contributions have yet to be jointly studied in individuals with GAD. This is surprising considering that many authors have recognized the need to study the nature of the relationship between intolerance of uncertainty and emotion regulation in GAD [25,51]. There are, however, two studies that have examined the combined effects of intolerance of uncertainty and emotion dysregulation in non-clinical individuals. In the first study, Ouellet, Langlois, Provencher, and Gosselin [52] used a cross-sectional design and found that having limited access to emotion regulation strategies partially mediates the relationship between intolerance of uncertainty and the tendency to worry. In the second study, Sahib, Chen, Cárdenas, Calear, and Wilson [53] used a six-week longitudinal design to show that emotion regulation strategies mediate the relationship between intolerance of uncertainty and anxiety in a community sample.

There is a gap in the current literature that needs to be empirically tested with adults who are diagnosed with GAD: do intolerance of uncertainty and emotion dysregulation each play a unique and significant role in the severity of GAD when both are examined simultaneously? If one of the two vulnerability factors appears to be more important than the other, clinicians should focus their efforts on the most important one. However, if the data reveal that both vulnerability factors are equally important, then each will require clinical attention during psychotherapy. Individual case conceptualization may reveal the need to prioritize one, but both would somehow deserve clinical attention.

Studying the joint and relative role of intolerance of uncertainty and emotion dysregulation in a clinical sample has important theoretical and clinical implications [20,51,52]. Comparing the contribution of each factor in a clinical sample is important, and examining their interaction would also document a potential multiplicative effect when people are both hypersensitive to uncertainty and unable to self-regulate emotions. In addition to providing additional empirical support for the multidimensional statistical model of Ouellet et al. [52] and Sahib et al. [53], the study of both intolerance of uncertainty and emotion dysregulation in a clinical sample could contribute to the development of novel psychotherapies for GAD [54].

The goal of the present study is to examine the respective roles of intolerance of uncertainty and emotion dysregulation in chronic worry and GAD severity in treatment seeking adults. It was postulated that, after controlling for the severity of depressive symptoms, intolerance of uncertainty and emotion dysregulation would each make a statistically significant and unique contribution to excessive worry (first hypothesis) and to the severity of GAD symptoms (second hypothesis). The study also examined, on an exploratory basis, whether the interaction between intolerance of uncertainty and emotion dysregulation would be a significant predictor of worry or GAD severity.

## 2. Materials and Methods

### 2.1. Participants

The sample for this study consisted of the 108 participants seeking treatment in the clinical trial by Bouchard et al. [27]. For the current study, complete data on intolerance of uncertainty and emotion dysregulation were collected at pretreatment. The sample, recruitment, and procedures are summarized below and described in detail in Bouchard et al. [27].

Participants were recruited in five university clinics specializing in the treatment of anxiety disorders across the Province of Québec (Canada). They were provided with cognitive behavioral therapy (CBT) for GAD based on the IUM [27], and completed the study questionnaires at pretreatment. The homogeneity of the sample must be acknowledged. The majority were women (see Table 1), which is consistent with epidemiological data showing that, compared to men, women are more likely to have GAD [55,56,57] and to seek psychotherapy services [58]. Only those with GAD, diagnosed using the Anxiety Disorders Interview Schedule for DSM-IV (ADIS-IV; [59]), were included in the study. Eligible and interested participants were informed in an interview of the implications of participating in the study. Informed consent was then obtained on a voluntary basis. All research and clinical interviews took place in private offices on campus sites. Confidentiality was guaranteed and protected in accordance with ethical requirements for psychotherapy and for research with humans (using an anonymized database, storing participant files in secure conditions, etc.). This study was part of a larger project that was approved by the ethics committees of all sites involved in accordance with the Canadian Tri-Council Policy Statement for Ethical Conduct for Research Involving Humans and the Helsinki Declaration.

The socio-demographic data of the current sample are reported in Table 1.

### 2.2. Assessment

The Penn State Worry Questionnaire [60] was used to measure excessive worry. The questionnaire includes 16 items rated on a 5-point Likert scale ranging from 1 (not at all typical of me) to 5 (very typical of me). A total score between 16 and 80 is obtained, where higher scores indicate greater levels of worry. This instrument has strong internal consistency (α = 0.95) [60].

The Worry and Anxiety Questionnaire [61] includes 11 items designed to provide a severity score for GAD symptoms according to the DSM-IV (and DSM-5). The items are scored on a 9-point Likert-type scale ranging from 0 to 8. A weighted total score of between 0 and 56 is obtained, with higher scores indicating more severe GAD. This questionnaire has good psychometric properties and is a sound measure of the severity of GAD [61].

The Intolerance of Uncertainty Scale [22] measures cognitive, emotional, and behavioral reactions to uncertainty. The IUS includes 27 items scored using a five-point Likert-type scale ranging from 1 (not at all characteristic of me) to 5 (entirely characteristic of me). A total score between 27 and 135 is obtained, where higher scores indicate greater intolerance to uncertainty. The questionnaire has good psychometric qualities, including excellent internal consistency (α = 0.95) [22], and evidence of convergent and divergent validity [28].

The Difficulties in Emotion Regulation Scale [32] is a questionnaire measuring six dimensions of emotion dysregulation: (a) lack of emotional acceptance; (b) difficulties engaging in goal-directed behavior in the presence of negative emotions; (c) difficulties controlling impulsive behavior; (d) lack of emotional awareness; (e) limited access to emotion regulation strategies; and (f) lack of emotional clarity. It contains 36 items scored on a five-point Likert scale ranging from 1 (almost never) to 5 (almost always). The total score ranges between 36 and 180 and a higher score indicates the presence of more severe difficulties in regulating emotions.

The Beck Depression Inventory (BDI) [62] is a questionnaire developed to measure depressive symptoms. To test our hypotheses, the BDI was used as a covariable. The questionnaire consists of 21 items rated from 0 to 3 and a total score between 0 and 63 is obtained. The higher the BDI score, the greater the severity of the depressive symptoms.

## 3. Results

Table 2 shows the intercorrelations among measures of worry, GAD severity, depressive symptoms, intolerance of uncertainty, and emotion dysregulation. All variables were significantly correlated with each other.

The first hierarchical regression tested whether intolerance of uncertainty and emotion dysregulation predicted excessive worry, after having entered depressive symptoms first in the regression model. This regression model was statistically significant [F_(3,107)_ = 13.19; *p* < 0.001] and explained 25.5% of the variance in excessive worry. The addition of the two predictors to depressive symptoms was statistically significant [F change _(2,104)_ = 15.55; *p* < 0.001], with both intolerance of uncertainty [95% CI (0.04, 0.17)] and emotion dysregulation [95% CI (0.04, 0.16)] playing a statistically significant role in the model (see Table 3).

To test the second hypothesis, a hierarchical regression was conducted to predict GAD severity controlling for the severity of depressive symptoms. In the first step, depressive symptoms were entered into the predictive model. In a second step, intolerance of uncertainty and emotion dysregulation were both entered simultaneously. The results indicate that the regression model was significant [F_(3,107)_ = 21.24; *p* < 0.001] and explained 38% (36% after adjusting for the number of parameters) of the variance in GAD symptom severity scores. Table 4 shows that adding both predictors in the second step explained an additional 10% of the variance in GAD severity [F change _(2,104)_ = 9.09; *p* < 0.001], and that intolerance of uncertainty [95% CI (0.01, 0.12)] and emotion dysregulation [95% CI (0.01, 0.12)] each had a significant and unique contribution to the predictive model beyond depressive symptoms [95% CI (0.09, 0.32)].

On an exploratory basis, the multiplicative effect of intolerance of uncertainty and emotion regulation was examined by entering their interaction in a third step following the second hierarchical steps reported in Table 3 and Table 4. The addition of the interaction between both predictors did not make a significant contribution to the predictive model for the severity of worry [F change _(1,103)_ = 0.05; Adjusted R-square change = 0.00; *p* = 0.82, ns], indicating that the joint multiplicative effect between intolerance of uncertainty and emotion dysregulation was not significantly related to the tendency to worry, as shown in Table 3 [95% CI (−0.003, 0.002)]. The addition of the interaction between both predictors did not make a significant contribution to the predictive model for the severity of GAD [F change _(1,103)_ = 1.17; Adjusted R-square change = 0.007; *p* = 0.28, ns], indicating that the joint multiplicative effect between intolerance of uncertainty and emotion dysregulation was not significantly related to GAD severity, as shown in Table 4 [95% CI (−0.003, 0.001)].

## 4. Discussion

The aim of the present study was to examine the relative contribution of intolerance of uncertainty and emotion dysregulation to worry and GAD severity in a clinical population of adults with GAD. It was postulated that, after controlling statistically for depressive symptom severity, higher scores on the measures of intolerance of uncertainty and emotion dysregulation would each make a unique and statistically significant contribution to excessive worry and to the severity of GAD. The presence of an interaction between intolerance of uncertainty and emotion dysregulation was also explored.

The study’s hypotheses were confirmed; intolerance of uncertainty and emotion dysregulation each played a significant role in the severity of worry and GAD. However, the interaction between the two factors did not make an additional contribution to either the tendency to worry or the severity of GAD. The unique contribution of the two core components of different theoretical models of GAD is noteworthy. As already mentioned by others [20,63,64], these models share conceptual similarities. The avoidance of uncomfortable internal experiences may play an explicit or implicit role in many models of GAD, including the IUM and the EDM [20]. But interestingly, our results show that perceiving uncertainty as threatening and negative emotions as dangerous/uncontrollable are distinct unique predictors. Although their interaction is correlated with worry and GAD severity, it appears that the interaction shares so much variance with each separate construct that its unique contribution is not statistically significant. This aligns with Behar et al.’s [63] suggestion, namely, that the IUM and the EDM may be complementary models, with the IUM focusing mainly on the appraisal of uncertainty per se as a threat [21,65] and the EDM focusing on emotions and their management [31]. Other studies have also documented complementarity among core features of different GAD models, with Deleurme, Parkinson, and Penney [66] documenting the unique roles of emotion dysregulation and negative beliefs about worry. Integrative theories are now being developed [52], but comprehensive models that include the features of many models have yet to be tested in a single study.

Our results suggest that intolerance of uncertainty and emotion dysregulation have distinct, additive effects on worry and GAD. However, there was no evidence of a multiplicative effect (interaction) of simultaneously being more intolerant of uncertainty and having more difficulties regulating emotions. In other words, each vulnerability does not impact the relation between the other vulnerability and worry/GAD. These results suggest that each vulnerability requires distinct clinical attention as improvement in intolerance of uncertainty may not necessarily lead to improvement in emotion regulation, and vice versa.

The current findings have significant clinical implications. Given the unique contributions of the vulnerability factors to the severity of worry and GAD symptoms, CBT techniques could be applied to focus simultaneously on both vulnerabilities. For example, a recently developed experiential treatment for GAD, Behavioral Experiments for Intolerance of Uncertainty [67], may offer an interesting avenue for individuals who are intolerant of uncertainty and also have difficulty regulating emotions. Behavioral experiments could be used to expose patients to uncertainty-inducing situations and encourage them to remain in the situation and let emotions emerge, and to sit with the uncomfortable feeling of not knowing. For example, a student with GAD may be encouraged to wait for 24 h before checking their exam result after it becomes available online. By being exposed to the uncertainty of not knowing for 24 h and embracing the elicited emotions, the student also learns to tolerate the uncomfortable emotions that are experienced during the waiting period. It is therefore possible to simultaneously address difficulties with uncertainty and with emotional regulation [49]. This focus on emotions while developing tolerance of uncertainty warrants further research.

Although this study has important strengths, such as the use of a clinical sample of adults with GAD, it also has certain limitations. For example, it was carried out on a homogeneous sample in terms of gender and culture. Although the gender distribution may be representative of people with GAD, it nevertheless limits our understanding of how our results may apply to males. It would also be interesting to replicate our study in cultural samples where emotions may be expressed differently. In addition, the majority of participants had at least one comorbid disorder. Although it is consistent with research showing that GAD is often comorbid with other disorders [7,68,69,70,71] and this is a strength in terms of generalizability, the presence of comorbid disorders may have influenced the results. Indeed, both intolerance of uncertainty and emotion dysregulation are also observed in other mental health problems [72,73]. This suggests that greater comorbidity may be associated with higher levels of intolerance of uncertainty and emotion dysregulation.

Further studies are needed, not only to replicate the results, but also to generalize them to other populations, including men and individuals from other cultures. In addition, future research could use longitudinal designs and experimental manipulations to examine the impact of changes in emotional regulation on intolerance of uncertainty, and vice versa. Also, further research is needed to enable us to better understand the mechanisms of interactions between IUM and EDM, should these exist. To this end, future research could investigate, as Ouellet et al. [52] and Sahib et al. [53] have done in community samples, whether emotion deregulation acts as a mediating variable in the relationship between intolerance of uncertainty and GAD severity. Most importantly, however, the model tested explained only 36% of the variance, suggesting that other variables in the IUM and the EDM may be involved in predicting GAD severity. Studies could simultaneously evaluate all components of the IUM and the EDM and use structural equation analyses to empirically document how certain components overlap or complement each other. Furthermore, since research indicates that temperament is associated with symptoms of certain anxiety disorders [74], future research could add this variable to the present predictive model in an attempt to better explain the severity of GAD.

## 5. Conclusions

The results of the present study indicate that higher levels of intolerance of uncertainty and more severe emotion dysregulation each make a unique contribution to the prediction of GAD symptom severity in a clinical population. These results have empirical, theoretical, and clinical implications. First, they provide additional empirical support for multidimensional models [52,53] and therefore may allow for the development of a coherent theoretical model of GAD that incorporates the IUM and the EDM. In addition, our results provide further empirical support for the relevance of developing integrative psychotherapies for GAD [54] that focus on uncertainty tolerance and emotion regulation.

## Figures and Tables

**Table 1:** Characteristics of the sample of 108 adults with generalized anxiety disorder seeking psychotherapy.

Variable	
Mean worry severity (SD) as measured by the PSWQ Mean GAD severity (SD) as measured by the WAQ	67.44 (6.69) 43.48 (6.29)
Mean depressive mood (SD) as measured by the BDI	20.77 (9.91)
Mean intolerance of uncertainty (SD) as measured by the IUS	87.10 (19.78)
Mean emotion dysregulation (SD) as measured by the DERS	106.14 (22.44)
Mean age (SD)	41.53 (15.86)
Sex (%) Women Men	88 (81.50) 20 (18.50)
White, non-Hispanic	100%
Annual self-reported income in Canadian dollars (%) Lower income (less than CAD 29,999) Low-middle income (CAD 30,000 to CAD 59,999) Middle-upper income (CAD 60,000 to CAD 89,999) Upper income (CAD 90,000 and more) Refused to answer	28 (25.90) 33 (30.60) 17 (15.70) 27 (25.00) 3 (2.80)
Employment status (%) Employed (full time or not) Unemployed (unemployed, retired, etc.)	68 (63) 40 (37)
Comorbid disorder (%) Anxiety disorder Mood disorder Anxiety and mood disorders Other disorder No comorbid disorder	24 (22.22) 8 (7.41) 10 (9.26) 25 (23.15)41 (37.96)

Note. PSWQ = Penn State Worry Questionnaire; GAD = generalized anxiety disorder; WAQ = Worry and Anxiety Questionnaire; BDI = Beck Depression Inventory; IUS = Intolerance of Uncertainty Scale; DERS = Difficulties in Emotion Regulation Scale.

**Table 2:** Bivariate Pearson correlations between all study variables (N = 108).

Variable	1	2	3	4	5	6
1. Excessive worry (PSWQ)		0.59 ***	0. 24 *	0.44 ***	0.45 ***	0.52 ***
2. GAD severity (WAQ)			0.52 ***	0.45 ***	0.48 ***	0.53 ***
3. Depressive mood (BDI)				0.39 ***	0.47 ***	0.50 ***
4. Intolerance of uncertainty (IUS)					0.44 ***	0.84 ***
5. Emotion dysregulation (DERS)						0.84 ***
6. Interaction between intolerance of uncertainty and emotion dysregulation						

Note. PSWQ = Penn State Worry Questionnaire; GAD = generalized anxiety disorder; WAQ = Worry and Anxiety Questionnaire; BDI = Beck Depression Inventory; IUS = Intolerance of Uncertainty Scale; DERS = Difficulties in Emotion Regulation Scale. * *p* < 0.05; *** *p* < 0.001.

**Table 3:** Hierarchical regression testing the individual contribution, and interaction, of intolerance of uncertainty and emotion dysregulation to the prediction of excessive worry after controlling for the severity of depressive symptoms (N = 108).

Predictors of Excessive Worry	Adj-R^2^	β	sr
Step 1 Depressive mood	0.05 *	0.24 **	0.24
Step 2 Depressive mood Intolerance of uncertainty Emotion dysregulation	0.26 ***	−0.03 0.31 ** 0.32 **	−0.03 0.27 0.27
Step 3	0.25 ***		
Depressive mood		−0.03	−0.03
Intolerance of uncertainty		0.39	0.09
Emotion dysregulation		0.40	0.10
Intolerance of uncertainty X emotion dysregulation		−0.13	−0.02

Note. Intolerance of uncertainty X emotion dysregulation = Interaction between intolerance of uncertainty and emotion dysregulation. * *p* < 0.025; ** *p* < 0.01; *** *p* < 0.001; Adj-R^2^ = Adjusted R-square; sr = semi-partial correlation (unique contribution).

**Table 4:** Hierarchical regression testing the individual contribution, and interaction, of intolerance of uncertainty and emotion dysregulation to the prediction of the severity of GAD after controlling for the severity of depressive symptoms (N = 108).

**Predictors of GAD Severity**	**Adj-R^2^**	**β**	**sr**
Step 1 Depressive mood	0.27 ***	0.52 ***	0.52
Step 2 Depressive mood Intolerance of uncertainty Emotion dysregulation	0.36 ***	0.33 *** 0.22 ** 0.23 **	0.28 0.19 0.19
Step 3 Depressive mood Intolerance of uncertainty Emotion dysregulation Intolerance of uncertainty X emotion dysregulation	0.36 ***	0.33 *** 0.56 0.56 −0.58	0.28 0.13 0.14 −0.08

Note. Intolerance of uncertainty X emotion dysregulation = Interaction between intolerance of uncertainty and emotion dysregulation. ** *p* < 0.01; *** *p* < 0.001; Adj-R^2^ = Adjusted R-square; sr = semi-partial correlation (unique contribution).

## Data Availability

Due to ethical considerations, the data are available only upon request when addressed to the Research Ethics Boards of the lead institution (comite.ethique@uqo.ca).
